# Structure-based inhibitor design of mutant RAS proteins—a paradigm shift

**DOI:** 10.1007/s10555-020-09914-6

**Published:** 2020-07-26

**Authors:** Kinga Nyíri, Gergely Koppány, Beáta G. Vértessy

**Affiliations:** 1 Department of Applied Biotechnology and Food Sciences, Budapest University of Technology and Economics, Budapest, 1111 Hungary; 2 Institute of Enzymology, Research Centre for Natural Sciences, Hungarian Academy of Sciences, Budapest, 1117 Hungary

**Keywords:** RAS proteins, GTPases, GAP, Drug target sites, Therapeutic strategies

## Abstract

As a member of small GTPase family, KRAS protein is a key physiological modulator of various cellular activities including proliferation. However, mutations of KRAS present in numerous cancer types, most frequently in pancreatic (> 60%), colorectal (> 40%), and lung cancers, drive oncogenic processes through overactivation of proliferation. The G12C mutation of KRAS protein is especially abundant in the case of these types of malignancies. Despite its key importance in human disease, KRAS was assumed to be non-druggable for a long time since the protein seemingly lacks potential drug-binding pockets except the nucleotide-binding site, which is difficult to be targeted due to the high affinity of KRAS for both GDP and GTP. Recently, a new approach broke the ice and provided evidence that upon covalent targeting of the G12C mutant KRAS, a highly dynamic pocket was revealed. This novel targeting is especially important since it serves with an inherent solution for drug selectivity. Based on these results, various structure-based drug design projects have been launched to develop selective KRAS mutant inhibitors. In addition to the covalent modification strategy mostly applicable for G12C mutation, different innovative solutions have been suggested for the other frequently occurring oncogenic G12 mutants. Here we summarize the latest advances of this field, provide perspectives for novel approaches, and highlight the special properties of KRAS, which might issue some new challenges.

**Electronic supplementary material:**

The online version of this article (10.1007/s10555-020-09914-6) contains supplementary material, which is available to authorized users.

## Introduction

**Fig. 1:**
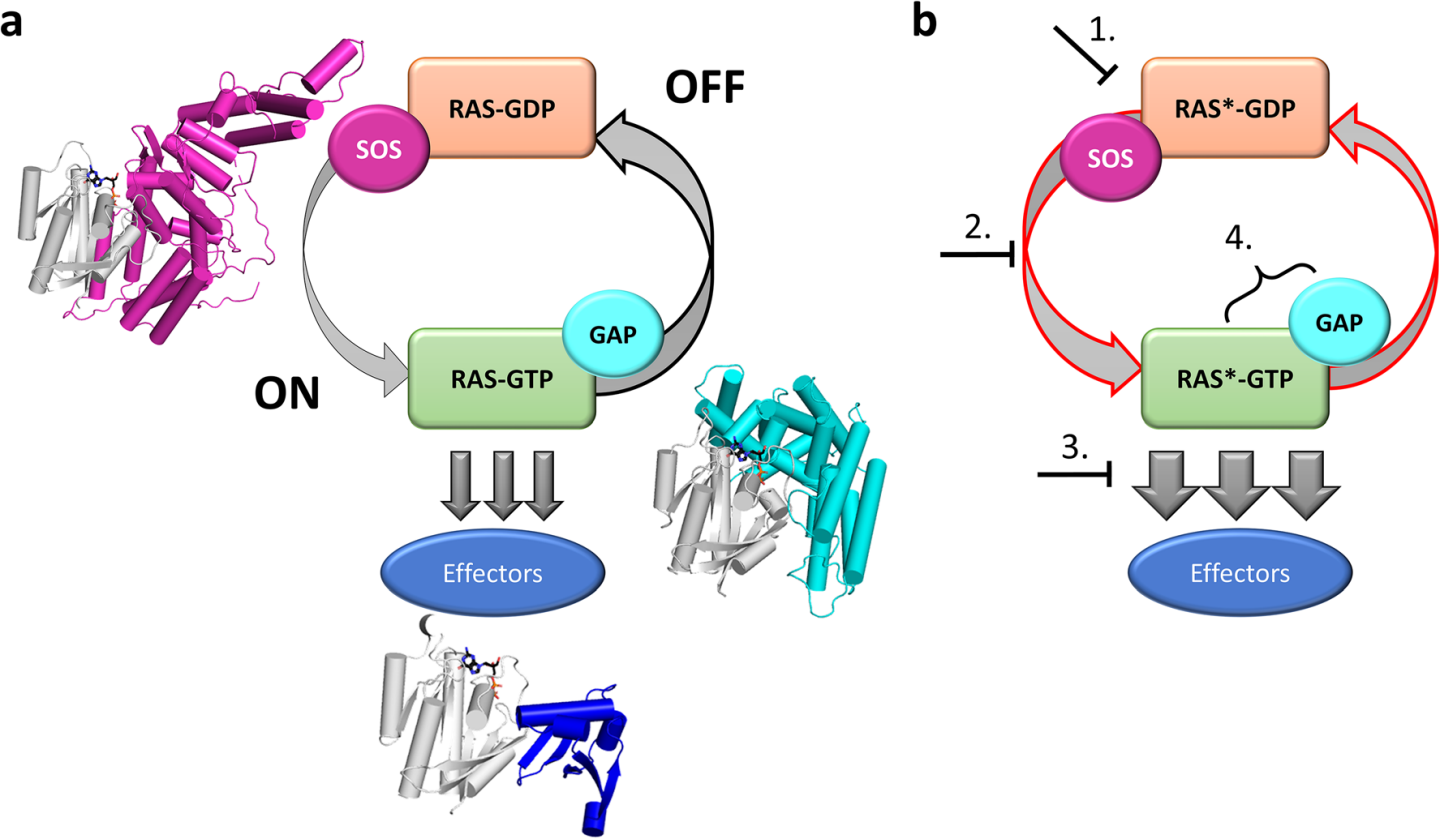
RAS signaling cycle and potential anti-RAS strategies. **a** Switching of RAS to active state happens through exchange of GDP to GTP aided by guanine nucleotide exchange factors (GEFs). In the GTP-bound state, RAS interacts with various effectors (RAF, PI3K, RALGEF, etc.). Decay of the signaling happens due to hydrolysis of GTP to GDP facilitated by GTPase-activating proteins (GAPs). **b** Mutations of RAS (*) perturbing its enzymatic activity and/or RAS-GAP complex formation lead to slower deactivation; the resulting enhanced signaling leads to oncogenic transformation of cells. To circumvent this imbalance, four potential mechanisms can be exploited. (1) Decrease SOS binding to reduce exchange of GDP to GTP. (2) Increase affinity to GDP over GTP (principally with covalent inhibitors). (3) Perturbation of effector binding to attenuate signaling. (4) Increase GAP binding of mutant RAS (applicable if GAP binds in competent conformation)

RAS proteins belong to the large family of small GTPases that are involved in numerous key physiological signal transduction processes reflecting widespread utilization of the same intriguing regulatory mechanism. In small GTPases, enzymatic hydrolysis of GTP and exchange of the product GDP to the next substrate molecule GTP is essentially coupled to two different regulatory factors: GAP (GTPase-activating protein) and GEF (guanine nucleotide exchange factor) (Fig. 1a). The facilitating action of GAP and GEF proteins are crucial in order to complete the enzymatic cycle since in the absence of these factors, the intrinsic catalytic rate constant (*k*_cat_) of the small GTPase enzyme is very low, and the release of the GDP product (characterized by the dissociation rate constant of the (small GTPase):GDP complex, *k*_d_) is also a slow process [1–3]. The substrate GTP-bound RAS protein serves as the conformational entity that is recognized by various signaling proteins (effectors) leading towards signaling cascades. GAP-assisted hydrolysis of GTP is required to switch off RAS to the GDP-bound enzyme conformer that is inactive in signaling. The exchange of GDP to GTP within the RAS substrate-binding pocket is practically not possible in the absence of the GEF factor binding to RAS:GDP (Fig. 1a). It is therefore crucial that both GAP and GEF proteins be available and be capable of binding to RAS as exactly such levels that is required for the actual status of cells and cellular needs for activation or inactivation of a specific signaling pathway [4]. Importantly, numerous different proteins can act as GAP or GEF or effectors in the different small GTPase-driven regulatory mechanisms. The cellular level of the nucleotides GTP and GDP usually do not constitute additional regulatory constraint since small GTPases in general and RAS proteins in particular are associated with very high affinities towards GTP and GDP (characteristic dissociation constant values, *K*_D_, are in the order of 100–0.1 nM to be compared with the usual cellular GTP, GDP concentration in the order of 10^−4^ M) [2]. This condition also results in the fact that RAS proteins are practically always present in their nucleotide-bound state: either as RAS:GTP (enzyme-substrate complex) or as RAS:GDP (enzyme product complex).

It comes as no surprise that this complex regulatory network harbors numerous protein sites where harmful mutations may perturb the correct process of events. Mutations in RAS proteins that interfere with productive functional binding of either GAP, GEF, or effector proteins can greatly perturb the sensitive modulatory machinery (Fig. 1b). Such mutations frequently lead to oncogenesis and as such, these constitute high biomedical concern and are in the focus of widespread research and drug development. In this respect, several mutations of the KRAS isoforms have been found to be frequently occurring in many types of cancer. Especially in cancers of the pancreas, colon, rectum, and lung, it is observed that several hotspot mutations can be identified at well-defined KRAS sites [5, 6]. Among these sites, the glycine 12 (G12) and glycine 13 positions often show mutations into cysteine, aspartate, and valine residues [6].

**Fig. 2:**
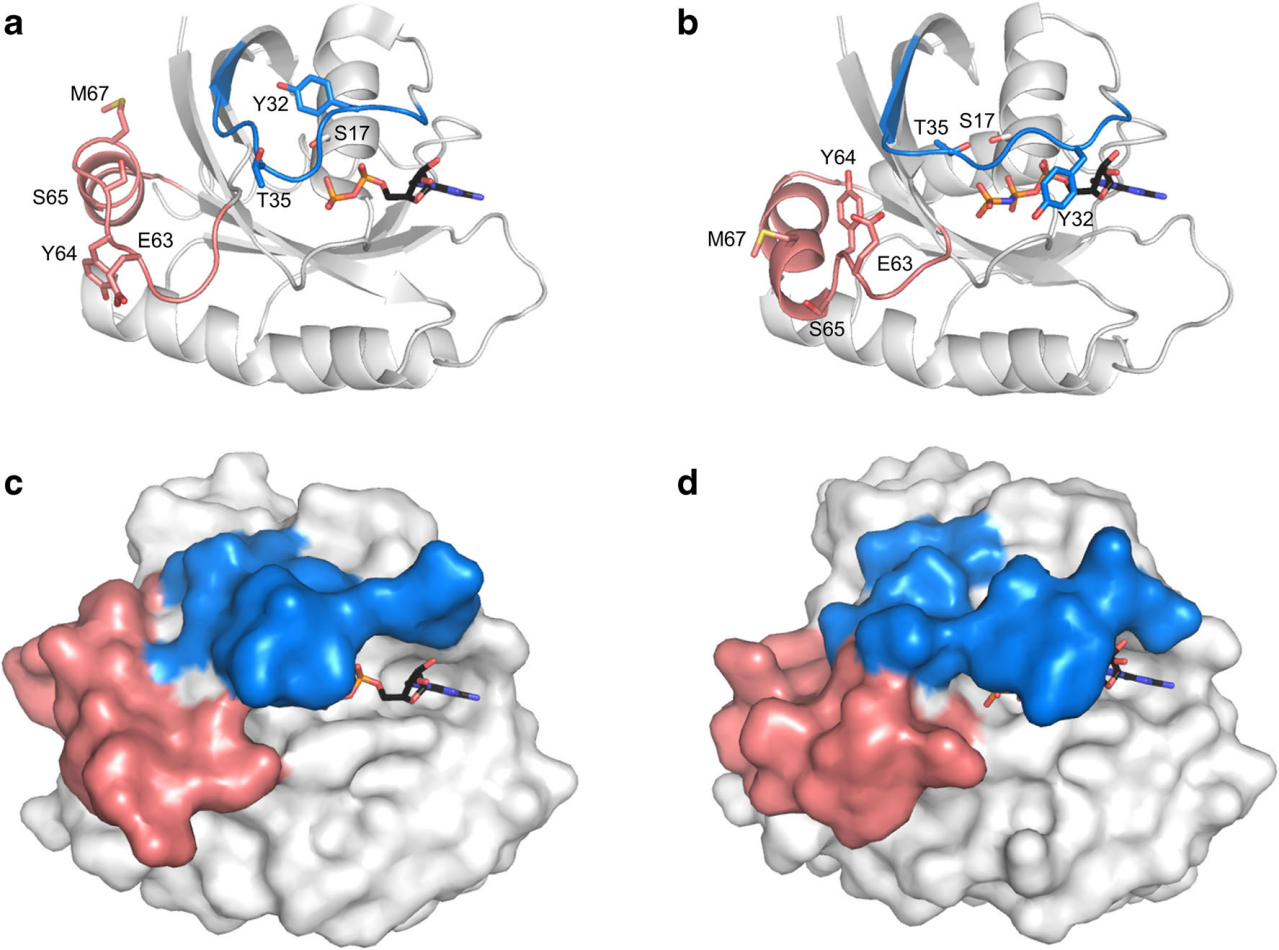
Conformational changes of RAS during signaling. The position of Switch-I (marine blue) and Switch-II (deep salmon) residues changes significantly between GDP (panels **a** and **c**, PDB ID: 4Q21) and GTP-bound states (panels **b** and **d**; PDB ID: 5P21). Nucleosides are shown as sticks with atomic coloring (carbon: black; oxygen: red; nitrogen: dark blue; phosphorus: orange). On panels **a** and **b**, residues showing large structural changes are represented as sticks with atomic coloring (C: variable; oxygen: red; nitrogen: dark blue; phosphorus: orange). Dramatic change of the protein surface between the inactive and active state is demonstrated on panels **c** and **d**. (Figure is designed based on Ref [7]). Figure was made by using PyMOL Molecular Graphics System

These mutations are termed as “activating mutations” due to the fact that they prevent functional interaction between KRAS and the GTP hydrolysis promoting GAP proteins, while they do not perturb GEF and effector binding. The structural basis of activation in the case of G12 position is that replacement of glycine with any other residue except proline interferes with GAP binding to KRAS through steric clashes with a key arginine residue of GAP [7, 8]. Hence, the mutations lead to the accumulation of the KRAS:GTP complex thereby overactivating the signal transduction pathways. Clinical approaches to restore normal functioning of KRAS aim to overcome the accumulation of the active KRAS:GTP complex by different means (Fig. 1b), discussed below in details. In these approaches, a significant paradigm shift occurred in the recent years that addressed the highly flexible and “moldable” character of the KRAS protein (Fig. 2).

Figure 2 highlights the quite substantial changes on the surface of the KRAS protein that are observed when comparing the KRAS:GTP (active in signaling) and KRAS:GDP (inactive in signaling) complexes. Flexibility is an inherent characteristic of KRAS since the large difference between the conformation of GDP- and GTP-bound states is the basis of allosteric function that transmits the signal from the enzyme active site to the surface segments involved in GAP, GEF, and effector binding. Due to flexibility, the position of switch regions is not well defined in several crystal structures or rendered only by crystal packing effects; this needed to be taken into account during interpretation of structures. For instance, despite the well-proven crucial role of Gln-61 in KRAS function [9], in most of the crystal structures of RAS with GTP or GTP analogues, it is rendered in a catalytically incompetent conformation, pointing outwards from the substrate-binding pocket. In addition to this, the physiologically relevant position of Tyr-32 (within Switch-I) in GTP-bound state might not have been deducible from crystal structures, due to its peculiar flexibility, formation of crystal contacts, and the potential rearrangement of water network upon cryo-cooling [10]. Thus, careful molecular modeling of the residues around the active site is necessary to gain an appropriate initial structure for inhibitor docking, during which flexibility should be taken into account.

The abovementioned structural ambiguities hinder also the revelation of the mechanism of GTP hydrolysis, which still lacks an unequivocal explanation despite being the key component of RAS function. Regarding the two aforementioned residues of special importance, it has been shown that Gln-61 does not act as a general base during hydrolysis, as it was erroneously assumed [11, 12]; instead, it has crucial indirect effect. It has been hypothesized that Gln-61 can contribute *via* positioning, but not activating, the catalytic water in the case of GAP-assisted hydrolysis, while in the absence of GAP Gln-61 may assist by positioning a second water potentially important in proton transfer to the γ-phosphate [13]. The role of Tyr-32 in hydrolysis is yet subject of scientific debate, the spectrum of interpretation of that extends from activation [14] even to interference [10]. Yet the exact mechanism of catalysis and thus the contribution of Gln-61 and Tyr-32 in particular have been elusive due to the astounding complexity of this apparently simple reaction. Deciphering of these issues is vital to promote successful inhibitor design and may pave the way for new approaches.

**Fig. 3:**
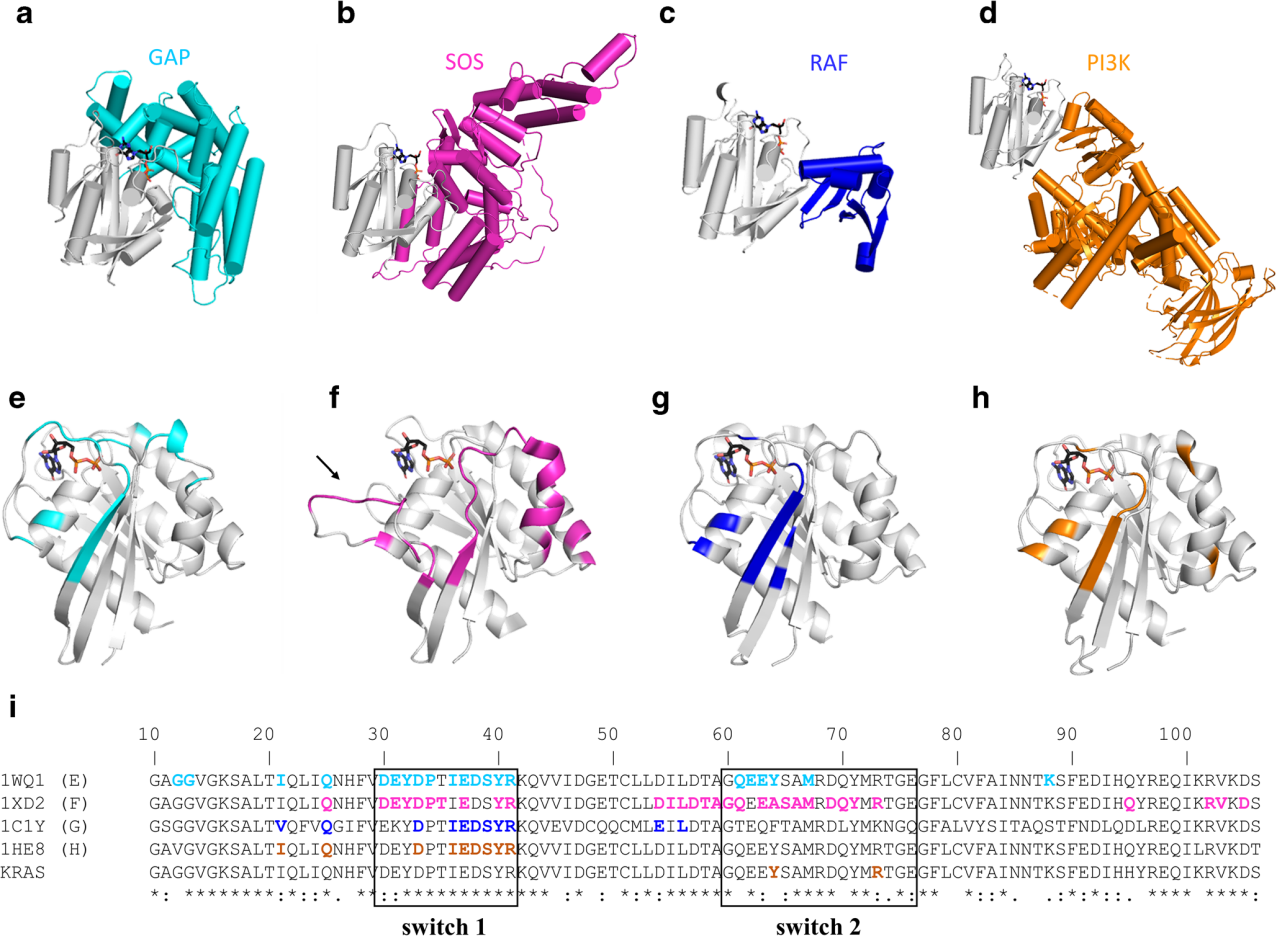
Protein-protein complexes and interaction surfaces of RAS. **a–d** Structures of RAS (gray) complexed with **a** GAP (cyan, PDB ID: 1WQ1), **b** GEF (magenta, PDB ID: 1XD2), **c** RAF-RBD (RAF-RAS-binding domain, dark blue, PDB ID: 1C1Y), **d** PI3K (orange, PDB ID: 1HE8). Proteins shown as cartoon. **e–h** Interaction surface of RAS (gray cartoon) with **e** GAP (cyan, PDB ID: 1WQ1), **f** GEF (magenta, PDB ID: 1XD2), **g** RAF-RBD (dark blue, PDB ID: 1C1Y), **h** PI3K (orange, PDB ID: 1HE8). To ease visualization of the nucleotide-binding pocket, GDP from RAS-GAP complex is shown in all structures as sticks with atomic coloring (carbon: black; oxygen: red; nitrogen: dark blue; phosphorus: orange). Black arrow on panel **f** points at Switch-I region, which undergoes large conformational changes upon RAS-GAP complex formation. **i** Sequence alignment of the RAS proteins shown in panels **a**–**h** with KRAS. Residues at the interaction surfaces are colored according to panels **e**–**h** respectively. Switch-I and Switch-II regions are boxed: Conformation of these two segments is significantly different in GDP- and GTP-bound structures enabling molecular recognition of the different states of RAS (*cf.* Fig. 2). Figure was made by using PyMOL Molecular Graphics System

Currently, the drug development projects follow one of four main strategies: (i) increase the level of GDP-bound protein over RAS:GTP (mostly with covalent inhibitors), (ii) perturb RAS:SOS complex formation to reduce exchange of GDP to GTP, (iii) disturb effector binding to attenuate signaling, (iv) enhance GAP binding of mutant RAS protein to decrease RAS:GTP level (applicable if GAP binding is possible in competent conformation) (Fig. 1b). We wish to point out that besides those mentioned above, diverse strategies to interfere with the oncogenicity of mutant RAS proteins have been suggested. For example, it has been proposed recently that agonists which facilitate apoptotic and autophagic cell death in mutant RAS cell lines can be applied [15]. Several anti-RAS strategies are based on upstream and downstream perturbation of the RAS cycle. These are out of the scope of this work; recent reviews on these strategies are available [16–18]. Below, we focus on attempts that target different surfaces of RAS and its major binding protein partners. Figure 3 shows in detail the RAS surfaces involved in protein-protein interactions.

## Different approaches and sites to target KRAS

Since it has been in the center of interest in oncotherapy for decades, there were many different approaches and strategies for targeting oncogenic RAS, with emphasis on the isoform that is most prevalent in cancer, namely KRAS.

**Fig. 4:**
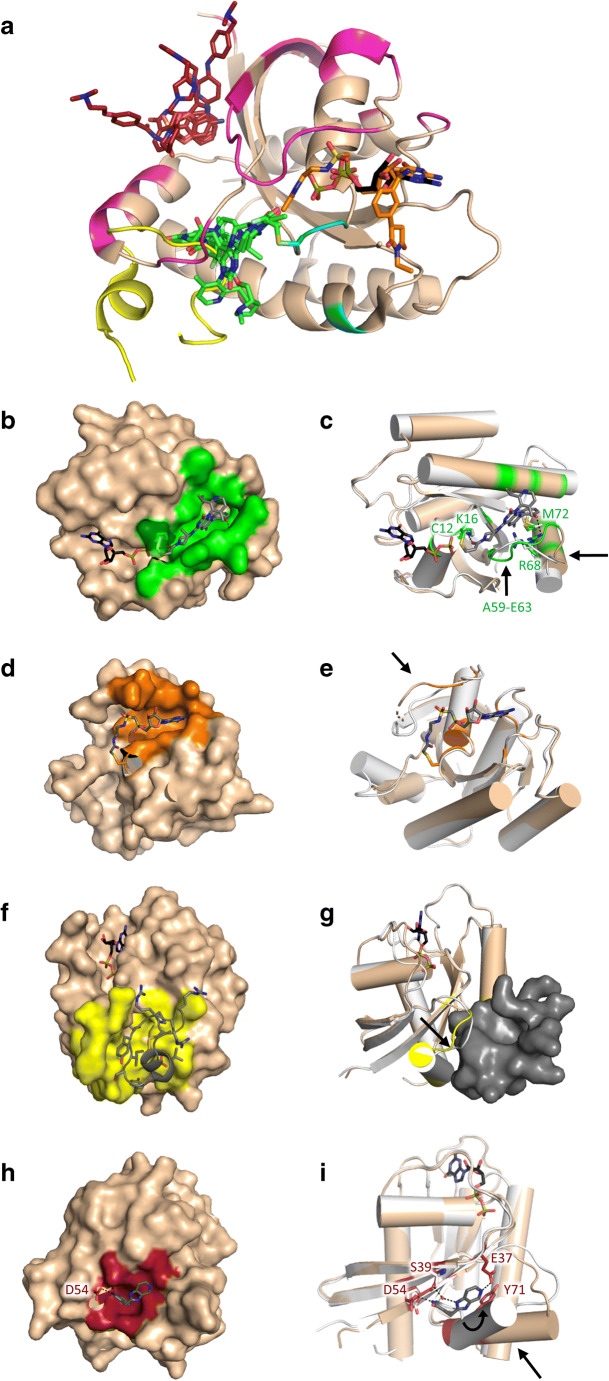
Binding sites of compounds designed to treat KRAS mutant cancers on KRAS protein. **a** Four main binding sites can be identified on the surface of KRAS. Representative examples of compounds perturbing KRAS function are shown as sticks or cartoon with atomic coloring (oxygen: red; nitrogen: blue; sulfur: yellow; phosphorus: deep olive; carbon: green, orange, yellow, and ruby representing the binding sites). Compounds shown (S12, S7, S3) are listed in Table 1 with bold lettering. KRAS (PDB ID: 5F2E) is shown as cartoon, magenta coloring represents the interaction surface of SOS and RAS (residues within 4 Å to SOS in SOS-bound structure PDB ID: 1XD2), cyan coloring represents part of the interaction surface of RAS and GAP (residues within 4 Å to GAP in GAP-bound structure PDB ID: 1WQ1), which is not shared with SOS-binding site. **b** Covalent inhibitor S7 (AMG-510, *cf* Table 1) binding to the Switch-II pocket is shown as sticks with atomic coloring (carbon: gray, other elements as above); G12C mutant KRAS is shown in surface representation, green coloring represents the binding site of the compound (residues within 4 Å to S7 PDB ID: 6OIM). **c** Superimposition of S7-bound KRAS (coloring according to panel **b**) with GDP-bound KRAS (white, PDB ID: 5W22) both shown as cartoons; black arrow points at the site of the most pronounced conformation change. Residues of wild-type and inhibitor-bound G12C mutant KRAS with significant importance in ligand binding are shown as sticks with atomic coloring (oxygen: red; nitrogen: blue; sulfur: yellow, white, and green respectively). **d** Inhibitor S12 (*cf* Table 1) binding to the nucleotide-binding site is shown as sticks with atomic coloring (carbon: gray, other elements as above); G12C mutant KRAS is shown in surface representation; orange coloring represents the binding site of the compound (residues within 4 Å to S12 PDB ID: 5KYK). **e** Superimposition of S12-bound G12C mutant KRAS (coloring according to panel **d**) with GDP-bound wild-type KRAS (white, PDB ID: 5W22) both shown as cartoons; black arrow points at the site of the most pronounced conformation change. **f** Peptide inhibitor binding to the Switch-II pocket is shown as cartoon and sticks with atomic coloring (carbon: gray, other elements as above); G12D mutant KRAS is shown in surface representation; yellow coloring represent the binding site of the compound (residues within 4 Å to the peptide PDB ID: 5XCO). **g** Superimposition of peptide-bound G12D mutant KRAS (coloring according to panel **f**) with GDP-bound wild-type KRAS (white, PDB ID: 5W22) both shown as cartoons. Peptide is in surface representation. Black arrow points at the site of the most pronounced conformation change. **h** Inhibitor S3 (*cf* Table 1) binding to the SI/II-pocket is shown as sticks with atomic coloring (carbon: gray, other elements as above); KRAS is shown in surface representation; ruby coloring represent the binding site of the compound (residues within 4 Å to S3 PDB ID: 4EPV). **i** Superimposition of S3-bound KRAS (coloring according to panel **h**) with GDP-bound KRAS (white, PDB ID: 5W22) both shown as cartoons; black arrow points at the site of the most pronounced conformation change. Residues of wild-type and inhibitor-bound KRAS with significant importance in ligand binding are shown as sticks with atomic coloring (oxygen: red; nitrogen: blue; sulfur: yellow, white, and ruby respectively).To ease following the orientation of the KRAS on the figures, GDP is shown as sticks in all but **d** and **e** panels with atomic coloring (carbon: black, other elements as above). See also Supplementary Fig. S1 for more details. Figure was made by using PyMOL Molecular Graphics System

The extremely low dissociation constant of RAS for GTP (with *K*_D_ in picomolar range) [19] and the high concentration of GTP [20] in cells makes competitive inhibition of GTP binding highly unlikely. Thus, attempts that aim to find other binding sites seem more promising. RAS proteins lack deeper clefts on their surface; however, due to the flexibility of the protein surface formation of several binding sites induced by compound binding has been observed (Fig. 4, see also Supplementary Fig. S1 for more details). The fluidity of RAS surface gives hope that new previously undiscovered binding sites can be identified, but presents difficulties for compound design through structure-activity relationship studies. The most evident approach is directly targeting the effector-binding region and disrupting RAS-GEF or RAS-effector interaction (*cf.* Fig. 3), thus counter-acting signal transduction. It is possible to inhibit effector binding by allosterically altering the Switch-I and Switch-II regions to an inactive conformation. This can be achieved by binding to a distant allosteric pocket or, in the case of the G12C mutant, targeting the nucleotide-binding site or a cleft nearby Switch-II with covalent inhibitors. Disruption of RAS activation is also achievable with the so-called pan-RAS inhibitors through targeting SOS-1, which is the most prevalent GEF of RAS proteins. However, despite the many promising strategies, so far, only four covalent G12C inhibitors and a KRAS-SOS1 inhibitor binding to SOS1 proved to be effective enough to get into clinical trials [21–26]. Structural details are available only for two of these candidates in clinical trials, namely AMG-510 and MRTX849, which are both covalent inhibitors [27, 28].

### Displacement of Switch-II with covalent inhibitors targeting the KRAS-G12C mutant

Two flexible binding sites are situated on the two sides of the α2-helix termed as SII-P and SI/II-pockets (Fig. 4b, c). SII-P is not present in the active form of KRAS and in the GDP-bound form, this pocket is reduced to a tight trench between α2- and α3-helices. The first molecules to target this site were inhibitors of KRAS G12C mutant that is most prevalent in non-small cell lung cancer and lung adenocarcinomas [29], in which presence of KRAS mutations mean worse prognosis, and elevated resistance for certain therapies [30–32]. Ostrem and colleagues presented compounds that bound covalently to the mutated Cys-12 residue and extend SII-P by adjusting the helix in Switch-II outwards that diminishes interaction with downstream effectors. Covalent inhibitors also reduce the affinity of KRAS G12C to GTP, thus preventing it to enter the active state [33].

These compounds are selective to KRAS in its GDP-bound form [34], for two separate reasons. One, the warhead responsible to form the covalent bond would sterically collide with the γ-phosphate of GTP, and two, in the active form, the SII-P pocket is unavailable due to the conformation of Switch-II (Fig. 4b, c). As GDP-bound KRAS exists at low levels in mutant cells, these compounds rely on the intrinsic GTPase activity of KRAS-G12C that is about half of the intrinsic activity of wild-type protein [35]. Development of such inhibitors needs unique approach since the activity of the most potent compounds is due to their KRAS-induced electrophilic reactivity towards Cys-12, while reversible affinity of those is weak [36, 37]. Thus, general methodology to enhance noncovalent binding affinity is not applicable in these cases, rather special electrophile warhead design [38] and covalent docking methods [39], which take into account the flexible surface of the binding site, are to be applied.

Taking a closer look on covalent inhibitors in complex with KRAS-G12C (PDB IDs: 5F2E, 5V9U, 6OIM, 6UT0) reveals the carbonyl group of the acrylamide warhead makes hydrogen bond to Lys-16, and a water coordinated by Mg^2+^ that are hydrogen bond partners of the γ-phosphate in GTP-bound state. Though there is great diversity in the scaffolds of the inhibitors, a common quality in all compounds is a hydrophobic moiety that fits into the hydrophobic pocket surrounded by Val-9, Met-72, Phe-78, Tyr-96, Ile-100, Val-103, and the carbon chain of Gln-99, while there are hydrophilic groups close to the loop of Switch-II that interact with Arg-68. There are additional hydrogen bonds with several other residues of Switch-II or α3-helix, depending on the compound. AMG-510 (S7 in Table 1), an inhibitor that is in clinical trials, stabilizes the GDP-bound state one step further by making a hydrogen bond with one of the oxygens of the β-phosphate of GDP (Fig 4 b, c). Compared to the GTP-bound conformation of Switch-II, the warhead of the inhibitors would collide with the loop of Switch-II; thus, residues Ala-59–Glu-63 of KRAS move away from the nucleotide-binding site. This dislocation affects Gly-60 as well, the residue that is responsible for connecting Switch-II to GTP. By binding to SII-P pocket, inhibitors push the α2-helix towards the main β-sheet, while Met-72 turns towards the inside of the protein [27]. Summing the previously discussed effects, it can be stated that covalent KRAS-G12C inhibitors achieve their effect by both a competitive (preventing GTP loading) and an allosteric (dislocating Switch-II) manner.

It is important to mention that the greatest advantage of this approach is that these inhibitors are specific to the G12C mutant of KRAS; thus, those have only slight cytotoxic effect on cells that have wild type or other mutant KRAS. The discovery of this allele-specific strategy led to the development of inhibitors of enhanced potency [34, 40], with elevated bioavailability [27, 28, 36, 41–44]. Several such drug candidates are subjected to clinical trials recently [21–24].

There were other strategies to covalently target Cys-12 of the G12C mutant, with covalent GTP analogues [45–47]. In this case, the guanosine mimetic inhibitors target the nucleotide-binding site of KRAS G12C, and bind to Cys-12 by a reactive warhead that replaced the γ-phosphate (Fig. 4d, e) [45, 47, 48]. These inhibitors have high affinity for KRAS G12C and bind efficiently to the nucleotide-binding pocket even in the presence of millimolar GTP and GDP that is equivalent to concentration in cells. It was shown that upon inhibitor binding, Switch-I and Switch-II are in the open, inactive conformation [47], and signal transduction was reduced as shown by depletion of pERK and pAKT levels. However, SML-8-73-1, the most potent of these inhibitors was prone to hydrolysis, at the phosphate-anhydride bond. To overcome this problem, several analogues were designed, but there was no success in identifying a compound that showed chemical stability and preserved high affinity for KRAS. It was suggested that the reason behind weakened activity is the loss of coordination between Mg^2+^ and the compound that is present in the case of natural guanosines [45]. Even if these difficulties can be solved, it is questionable whether this approach will be viable *in vivo* as there are many potential off-target activities, due to the vast number of GTP-binding proteins in the cellular milieu.

The success of covalent inhibitors of KRAS G12C suggested that the same strategy might be viable for G12D and G13D mutants as well, since the carboxyl group of aspartate can react to functional groups such as, aziridine, or chloroacetamide [49–51]. However, it was shown that in the case of KRAS, compounds that have electrophile groups that would be appropriate for aspartate engagement cannot efficiently label the G12D mutant [52], the possible reason being that Asp-12 is arranged in a way that cannot be attacked by compounds that bind into the SII-P pocket. However, there are results that show promising approaches of specific inhibition of other oncogenic KRAS mutants, besides G12C.

Peptides can also target the cleft between α2- and α3-helices as it was shown by Sakamoto et al., who reported that KRpep-2, a cyclic peptide, binds to the G12D mutant of KRAS with relative selectivity towards G12D mutant over wild-type and G12C KRAS [53]. The peptide forms several hydrogen bonds and hydrophobic interactions with residues in both α2- and α3-helices, and stabilizes Switch-II in a conformation that is similar to the GDP-bound inactive state (Fig. 4f, g). A likely structural reason behind G12D selectivity is that Asp-12 of G12D can form a hydrogen bond with Gln-61, and stabilize Switch-II in a conformation which is suitable for KRpep-2 binding [54].

### Targeting protein-protein interaction surface with small molecules

Within the effector interacting region of RAS, the loci that are the most targetable by small molecule compounds involve the SI/II-pocket, the trench between α2-helix, and the main β-sheet (β1-β3) of RAS (Fig. 4h, i). Crystal structures demonstrate the flexibility of the SI/II-pocket, as upon compound binding the pocket can be extended by the rotation of Asp-54 and Arg-41 away from the binding site, while preserving the salt bridge between the two side chains, as it was first shown by Maurer et al., who reported small indole- and benzamidine-based compounds that inhibit the formation of RAS-SOS complex upon binding into SI/II-pocket [55]. Though the pocket is absent in GDP-bound state of RAS and only becomes visible in the GTP-bound state, the molecules that target this site show little preference for GTP-bound RAS and are able to engage the GDP-bound state as well [56]. In GDP-bound state, Tyr-71 of KRAS forms hydrogen bond with Asp-54 and Ser-39, but upon compound binding, it tilts away from the pocket to a position that is similar to the GTP-bound state, while Met-67 turns away to open a shallower cleft (Fig. 4i). This results in the slight displacement of the α2-helix and Switch-II [57], and the compound in the pocket prevents Tyr-71 to align into the hydrophobic core of SOS and to form hydrogen bond with Tyr-910 of SOS [58]. In the case of the indole derivative S3 (compound 4 in ref. [57], *cf.* Table 1), the indole ring fits into the hydrophobic pocket that was previously occupied by the side chain of Tyr-71, while there are hydrogen bonds formed between Asp-54 and the indole ring and Glu-37 and the imidazopyridine group of S3, whereas the latter is connected to Ser-39 through a water bridge (Fig. 4i).

**Table 1:** Comparative table of recent KRAS targeting strategies. Molecules that are in bold, are shown in Fig. 4 and Supplementary Figure S2, PDB IDs containing those molecules are shown in bold as well

Binding target/site	Effect	Compound type	PDB ID	Representative example	Reference
SI/II-pocket (α2-helix and β1-β3 sheet) in the PPI surface	Inhibition of GEF, GAP, and effector interaction	Small molecule	6GJ5-**6GJ8**	**S1-BI-2852**	Kessler-2019 [56]
	GEF-mediated nucleotide exchange inhibition		4DSO, 4DST, **4DSU**	**S2-Benzimidazole** DCAI	Mauer-2012 [55]
	Inhibition of RAS-SOS binding		4EPR, 4EPY, 4EPX, 4EPW, 4EPT, **4EPV**	**S3–‘Compound-4’**	Sun-2012 [57]
	Inhibition of effector interaction		5OCO, 5OCT, 5OCG, **6FA1**, 6FA2, 6FA3, **6FA4**	**S4-ABD-4** **S5-ABD-7**	Qevedo-2018 [60]
			6GOD, 6GOE, 6GOF, 6GOG, 6GOM, 6GQT, 6GQW, 6GQX, 6GQY	Ch-3	Cruz-Migoni-2019 [59]
Switch-II pocket (S-IIp)	Inhibition of GTP loading to RAS	Covalent small molecule	**5F2E**	**S6-ARS-853**	Patricelli-2016 [34]
			5V9U	ARS-1620	Janes-2018 [42]
			**6OIM**	**S7-AMG-510**	Canon-2019 [27]
			**6UT0**	**S8-MRTX849**	Fell-2020 [28]
P110 pocket on the allosteric lobe of KRAS	Allosteric inhibition of effector interaction	Small molecule	**–**	KAL-21404358	Feng-2019 [67]
Allosteric lobe of KRAS	Competitive inhibition of GEF	Antibody-like protein	6H46, 6H47	DARPin K13, DARPin K19	Bery-2019 [68]
Hydrophilic pocket in CDC25 domain of SOS	SOS-mediated nucleotide exchange overactivation, biphasic modulation of ERK pathway, through inducing negative feedback	Small molecule	4NYI, 4NYJ, 4NYM	–	Burns-2014 [81]
			6D5W, 6D5V, 6D5M, 6D5L, 6D5J, 6D5G 6D5H, 6D5E, 6D59, 6D56, 6D55	‘Compound-34,’ ‘Compound-65’	Hodges-2018 [72]
	Inhibition of SOS mediated RAS activation.	Small molecule	6EPL, 6EPM, 6EPN, 6EPO, 6EPP **5OVI**	**S9 BAY-293**	Hillig-2019 [73]
					Evelyn-2014 [74]
	Inhibition of SOS-mediated RAS activation	Small molecule	4URU, 4URV, 4URW, 4URX, 4URX, 4URY, **4URZ**, 4US0, 4US1, **4US2**	**S10, S11**	Winter-2015 [75]
RAS-SOS interface	Stabilizing RAS-SOS complex				
Covalent bond with C118 of RAS near guanosine binding site	Disrupting nucleotide binging, disruption of effector binding	Covalent small molecule			
Nucleotide binding site	Disrupting effector interaction	Covalent guanosine analogue	**5KYK**	**S12** **XY-02-075**	Xiong-2017 [45]
PPI surface	Binding to PPI in GTP-bound state, obstructing effector interaction	Antibody	–	–	Shin-2017 [64]
		Antibody-like protein	5O2S, 5O2T	DARPin K27, DARPin K55	Guillard-2017 [62]
Switch-II pocket (S-IIp)	Inhibition of RAS-SOS complex	Peptide	5XCO	RT11	Sogabe-2017 [54]
RAS-GAP interface	Promoting RAS-GAP interaction	Small molecule	–	**S13**	Nyíri-2020 [83]

The changes within Switch-II conformation do not seem significant enough to effectively inhibit SOS and effector interaction; accordingly Cruz-Migoni et al. reported compounds, identified by surface plasmon resonance screening, that show no inhibitory effect despite displacing several residues of RAS that contribute to RAS-SOS binding [59]. In an earlier work by the same group, the crystal structure of compound-bound RAS was superimposed with complexes of RAS and its downstream effectors (RAF, PI3K, and RALGDS) and it was shown that compound Abd-7 would collide with each interacting partner [60]. However, when cross-over compounds were designed by combining the binding region of the biologically inactive compounds with the part of Abd-7 that reaches out into the protein-protein interaction (PPI) surface, inhibitory effect was observed [59]. Thus, it was concluded that these compounds achieve their inhibitory effect through sterically colliding with SOS and downstream effectors. This was further proven by *in vitro* assays that monitored SOS-mediated activation, as well as by cell-based assays in which inhibition of downstream signaling was observed in the presence of the most effective compounds, while compound treatment also decreased cell viability in micromolar concentrations [56, 59, 60]. An additional inhibitory mechanism of these molecules can be the induction of non-functional dimer formation of KRAS, like in the case of BI-2852 [61]. It is worth to mention that smaller antibodies and antibody-like proteins can also target the PPI of RAS, and compete with effector binding. Expression of DARPin K55 and RT11 in KRAS mutant cells could effectively dampen downstream signaling, and reduce cell viability, but despite the nanomolar dissociation values, their therapeutic *in vivo* effect was still low, due to troubled intracellular engagement [62–64]. Perturbation of effector binding of GTP-bound KRAS could also be achieved by small molecules, which drive the formation of a ternary complex with cyclophilin A according to a recent report [65]. Correspondingly, compounds that block the HRAS:RAF and simultaneously the HRAS:SOS interaction have been recently reported; the same approach could also be exploited against KRAS [66].

### Allosteric rearrangement of Switch regions through binding to a distant site

Another approach that was able to achieve selectivity towards G12D employs small molecules that bind to the P110 pocket on the allosteric lobe. This pocket is surrounded by α5-helix, the loop between α3-helix and β5-sheet, and the C-terminal of α4-helix. Interestingly, despite the high sequence similarity, this pocket is less prevalent in the case of HRAS and NRAS, resulting in an optimal target for KRAS-specific inhibition. Feng and colleagues presented a series of quinoline- and piperazine-based molecules that bind to this site. NMR results showed that upon binding of KAL-21404358, an early compound hit, Switch-I (Asp-33, Ser-39), and Switch-II (Leu-56, Gly-60, Met-67, Thr-74 and Gly-75) undergo conformational changes, suggesting an allosteric effect on KRAS-G12D. This resulted in inhibition of RAS-Raf interaction, and weak depletion of phosphorylated Akt, and ERK within treated cell lines [67]. These examples show that while selectively targeting non-G12C mutants of KRAS is complicated and requires less-straightforward strategies, it is possible to achieve, by taking advantage of the small structural changes that are caused by the mutated residue.

Antibody-like DARPin macromolecules can also target the allosteric site of KRAS, more accurately the interface containing α3-helix, loop 7, and α4-helix. Within α3-helix, there are several residues that are not conserved among the isoforms that can be exploited for isoform-specific engagement. NMR data suggests that there are no significant conformational changes in either of the Switch regions caused by macromolecules binding to the allosteric lobe; rather, these macromolecules disrupt KRAS dimerization and inhibit nucleotide exchange by colliding with SOS [68].

### Targeting KRAS function through small molecules that bind to SOS

The SOS-RAS interaction, with SOS being the most important GEF of RAS, is an obvious target for inhibition, especially that certain oncogenic mutants still rely on upstream activation [34, 35]. Moreover, since oncogenic KRAS can increase the activation of wild-type isoforms as well, through positive feedback by binding to an allosteric regulatory site on SOS [69, 70], disruption of this step in signal transduction can prove to be effective.

To perturb this interaction with the so-called pan-RAS inhibitors, the surface of the SOS protein is just as viable target, as that of RAS, as it was shown by several groups [71–74]. Winter et al. were able to identify three distinct pockets on HRAS-SOS complex, one at SOS CDC25 domain, one at the HRAS-SOS interface, and one covalent binding site on HRAS. Though they were unsuccessful in showing biological activity in the first two cases, they reported inhibition of RAS-SOS function in the case of covalent compounds that binds to Cys-118 close to the nucleotide-binding site [75]. Later however a different group presented small-molecules that bind to the same pocket on SOS CDC25 domain, and have inhibitory effect on the RAS-SOS interaction [73].

**Fig. 5:**
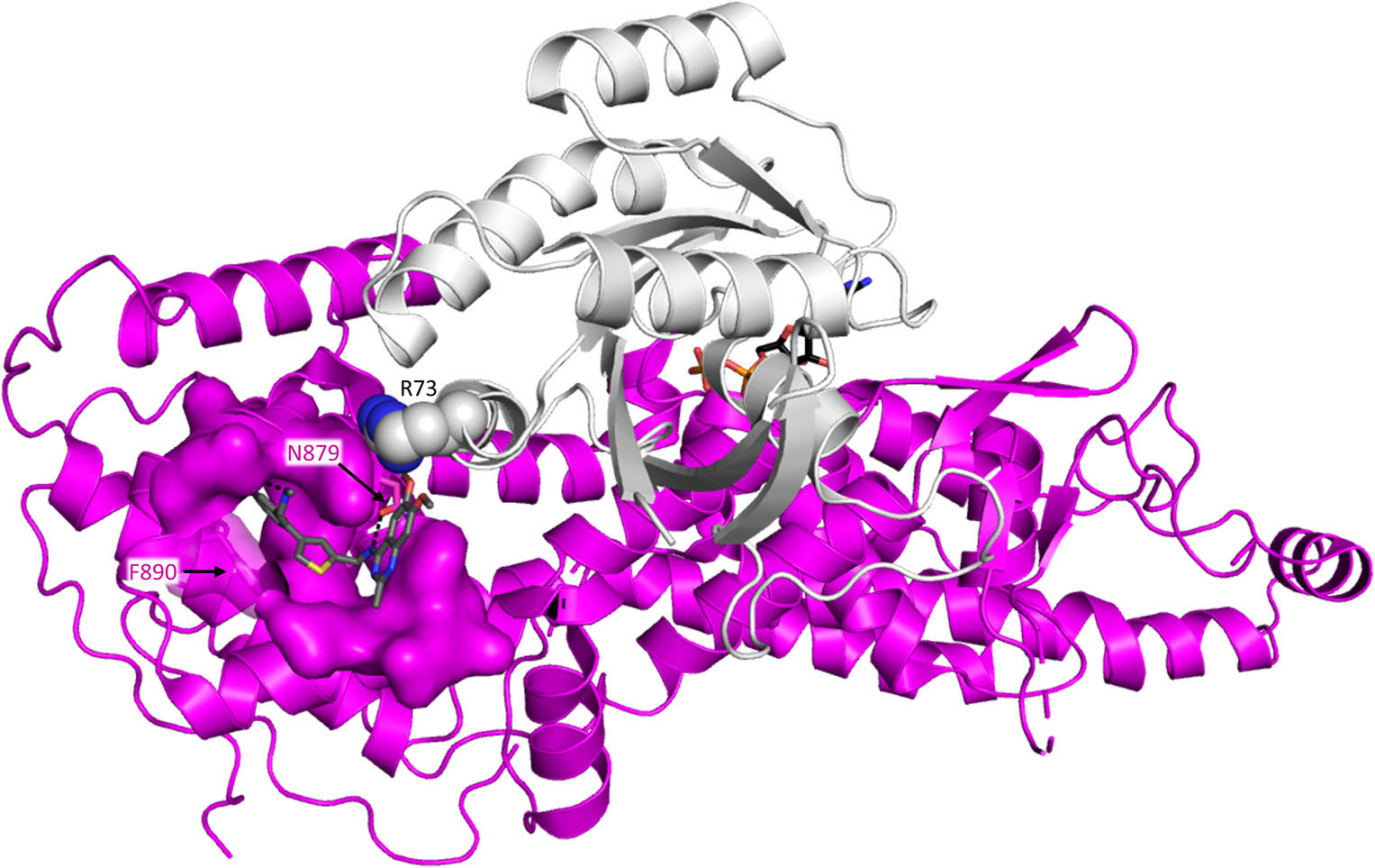
Binding site of compounds perturbing KRAS-SOS interaction on the surface of SOS. S9-bound SOS (PDB ID: 5OVI) is shown as magenta cartoon; surface of residues within 4 Å to S9 is shown to visualize the binding site (see Table 1 for definition of S9). S9 and GDP are shown as sticks with atomic coloring (oxygen: red; nitrogen: blue; sulfur: yellow; phosphorus: orange; carbon: gray and black, respectively). Residues of key importance in S9 binding of SOS (Asn-879 and Phe-890) are shown as sticks. To visualize the site of interference of S9 with KRAS binding, side chain of Arg-73 is shown as spheres with atomic coloring (carbon: white; nitrogen: dark blue); position of KRAS (white cartoon) is determined by the overlay of SOS-KRAS complex (PDB ID: 1XD2) to S9-bound SOS. Figure was made by using PyMOL Molecular Graphics System

The pocket on the SOS surface is surrounded by two longer (α46, α49) and two short (α49,α48) helices, and faces towards the C-terminal end of Switch-II of HRAS (Fig. 5). It is surrounded by three aromatic residues that are capable of making hydrophobic interactions with the aromatic scaffolds of the compounds. In several structures of SOS in complex with compounds, Phe-890 is turned outward from the bottom of the pocket (Phe-out conformation), further deepening that and presenting an optimal partner of π-π stacking interactions. The most potent inhibitor of this series, BAY-923, takes up the deeper pocket with its phenyl and thiophene moiety. The phenyl group establishes hydrophilic interaction with Phe-890, in the Phe-out formation, while the quinazoline moiety of the compound fits into a shallower sub-pocket, between the aromatic rings of Tyr-884 and His-905 and forms π-π stacking interaction with those [73]. The inhibitory effect is achieved through stabilizing Tyr-884 in a conformation that is turned away from Arg-73 of KRAS, weakening the interaction between the two protein surfaces. Compound binding alters the conformation of Asn-879 and Ser-881 that would form hydrogen bonds with Arg-73 and Asp-69 residues of KRAS. Additionally, the methyl-ether groups of the quinazoline ring would likely collide with the carbon chain of Arg-73 of KRAS further contributing to the disruption of the hydrogen bond between Arg-73 and the backbone of Asn-879 (Fig. 5).

There were reports of molecules that bind to this same pocket on SOS, but instead of inhibiting nucleotide exchange, they activate SOS [71, 72] (*cf* Supplementary Fig. S3). Though this is seemingly exactly the opposite of the desired effect, this approach has some advantages, as activated RAS can trigger cell death [76]. Cells only tolerate overactivation below a certain threshold, over which defensive pathways are activated, and apoptosis is induced. There is evidence to suggest that the reason behind KRAS being the most oncogenic of the RAS isoforms is its lower quantity in cells, due to rare codons in *KRAS* genes. Elevated levels of cellular KRAS as a result of codon optimization showed reduced tumor burden in mice [77, 78]. Expression of HRAS-G12V in non RAS-dependent human cancer cells induced caspase-independent cell death [79], and activation of RAS *via* chemotherapeutic agents can induce apoptosis [80]. This overactivation approach thus might be more robust compared to RAS inhibition, as treatment with RAS inactivators can be avoided by rescue pathways (*cf* Section 3).

Activating compounds were further optimized by structure activity relationship studies. During optimization of an early hit (PDB ID: 4NYM), one of the indole rings that fits into the deeper part of the pocket formed on the surface of SOS is replaced with a N3-benzyl substituted benzimidazole ring, while the other indole moiety is removed resulting in a compound with enhanced properties (PDB ID: 6D5G) [72, 81] (*cf* Supplementary Fig. S3). This positions Phe-890 into the “Phe-out” conformation, which takes up the space that was previously occupied by the indole group that was removed, while the benzyl ring and hydrophilic substituents fill the sub-pocket under Phe-890. This alteration of SOS side chain does not disturb RAS-SOS complexation. A chloride-substituent is present at the hydrophilic pocket under His-905, and hydrogen bonds are formed between the tetrahydropyridine group substituted at C-7 and Glu-902, and between the piperazine ring and Asp-887.

The best hits of Hodges et al. showed higher affinity and demonstrated a robust biphasic deactivation of the ERK pathway. Low micromolar (10–30 μM) treatment with compounds (**42**, **64**) increased RAS-GTP levels linearly, while pERK levels showed increase at up to 1 μM compound concentration and decrease at higher compound concentration. However, it has not been assessed whether the compounds have an effect on cell viability [72].

It is intriguing that compounds that bind to the same location can have exactly the opposite effect. Comparing the crystal structure RAS-SOS complex bound to inhibiting (PDB ID: 5OVI) and activating (PDB IDs: 6D6G, 6D56, 5WFR) compounds, it becomes clear that activating compounds do not, or only slightly, alter the conformation of residues compared to untreated RAS-SOS complex (PDB ID: 1XD2). While the inhibiting BAY-293 molecule causes several changes in SOS CDC25 domain that can cause the disruption of SOS-KRAS complexation, the only evident alteration of activator-bound SOS from the untreated structure is the side chain of Phe-980 being in the “Phe-out” conformation; however, this is relatively far from RAS-binding surface of SOS, and thus likely does not play a key role in the RAS-SOS interaction. Hence, it is probable that activating compounds are effective through stabilizing the CDC25 domain of SOS in a conformation that is optimal for RAS binding [71, 72, 82].

### Restoring KRAS function through GAP binding

Finally, we refer a newly identified family of molecules that may enhance the interaction between the KRAS G12D mutant protein and GAP [68]. This approach aims at stabilizing the KRAS-GAP complex to prevent GEF and effector interactions, thus inhibiting downstream signaling.

**Fig. 6:**
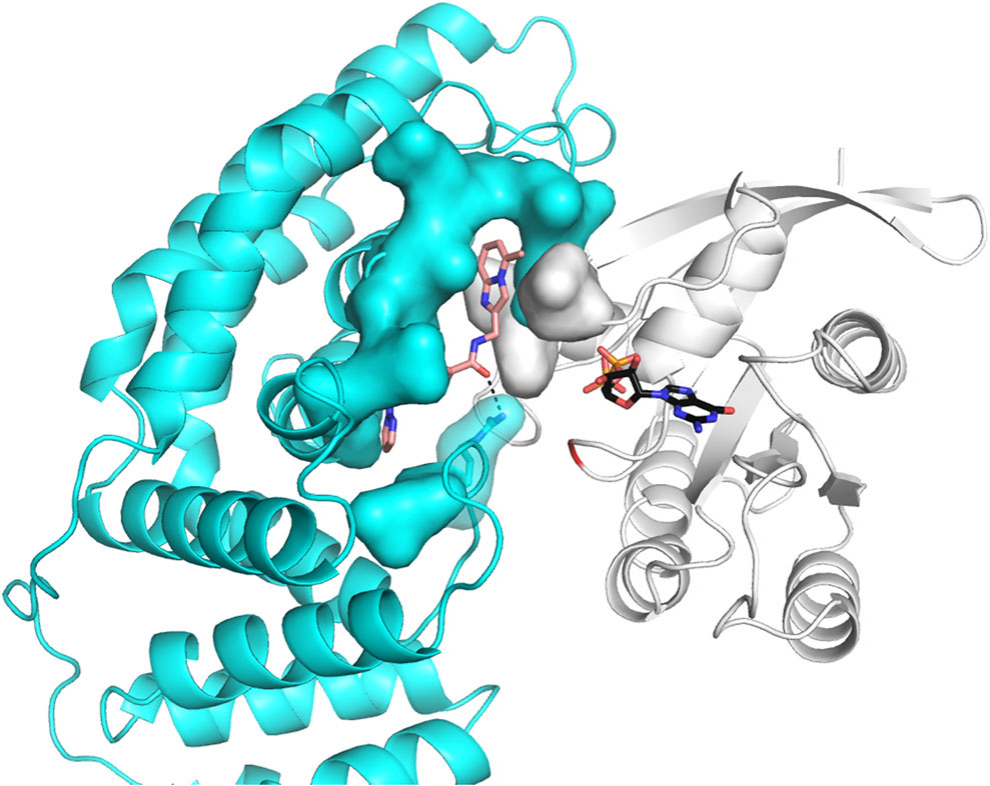
A compound promoting interaction between mutant KRAS and GAP. Model of S13 (*cf* Table 1) bound to GAP-KRAS-G12D complex is shown as sticks with atomic coloring (carbon: salmon; oxygen: red; nitrogen: blue; sulfur: yellow; phosphorus: orange). GDP is shown as sticks with atomic coloring (carbon: black, other elements as above). GAP is shown as cyan cartoon; KRAS is represented as white cartoon. Surface of residues interacting with S13 is shown. Dashed black line represents hydrogen bonding. Figure was made by using PyMOL Molecular Graphics System

Compound binding was verified by *in silico* modeling, where S13 binds to the KRAS-GAP interface between Switch-I of KRAS and the turn between α19- and α20-helices of GAP, while reaching into a small pocket of GAP between α17-helix and a turn motif consisting of residues 785–789 (Fig. 6). To experimentally verify structural interaction between the new molecules and the KRAS-GAP complex, crystallization trials are in progress. The efficacy of such small molecules are demonstrated in human cancer cell cultures, where this compound inhibits the proliferation of cells containing the KRAS G12D allele with some preference [83].

## Adaptive response hinders the effectivity of KRAS inhibitors

Although there is a significant advance in the research of KRAS inhibitors, there are still many problems to be solved to finally develop an effective therapy for KRAS mutant cancers. For instance, the currently best response for G12C mutant specific inhibitors was partial response in patients with lung cancer and stable disease (no partial response) in the case of colorectal and other solid tumors [21, 27, 84, 85]. In the case of ARS-1620 treatment, this is likely to happen due to adaptive responses *via* facilitated upstream (EGFR) and downstream (CRAF) signaling which could not be avoided even with continuous drug treatment [86]. In addition, evidence shows that anti-KRAS treatment frequently activate adaptive resistance mechanisms that enable cell survival *via* suppression downstream mitogen-activated protein kinase (MAPK) signaling or directly the expression of KRAS [87].

A potential way to overcome adaptive response is combination therapy [88, 89]. A prominent example is combination of covalent KRAS G12C inhibitor MRTX849 with EGFR, mTOR, or SHP2 inhibitors which were shown to be more effective than monotherapy in tumor models [90]. It has also been proposed that targeting effector binding of GTP-bound KRAS *via* ternary complex formation with cyclophilin A can overcome resistance driven by enhanced upstream signaling [91].

We conclude that the exploitation of hidden binding sites on KRAS protein opened the way to overcome the “non-druggable” paradigm and led to many new developments based on a variety of drug candidate compounds. Since several drug binding sites on the surface of KRAS are not apparent in the absence of the compounds, the structure-based approaches relying on drug-free KRAS structures need to be used with caution and have to be optimally complemented by phenotypic high-throughput screens in cellular studies.

## Electronic supplementary material

ESM 1 (PDF 518 kb)

## References

[1] Wittinghofer A (2016). GTP and ATP hydrolysis in biology. Biopolymers.

[2] John J, Sohmen R, Feuerstein J, Linke R, Wittinghofer A, Goody RS (1990). Kinetics of interaction of nucleotides with nucleotide-free H-ras p21. Biochemistry.

[3] Herrmann C, Martin GA, Wittinghofer A (1995). Quantitative analysis of the complex between p21 and the Ras-binding domain of the human Raf-1 protein kinase. Journal of Biological Chemistry.

[4] Stalnecker, C. A., & Der, C. J. (2020). RAS , wanted dead or alive : advances in targeting RAS mutant cancers. Science Signaling, Stalnecker, 1–7. 10.1126/scisignal.aay6013 PMC7393681 32209699

[5] Khan AQ, Kuttikrishnan S, Siveen KS, Prabhu KS, Shanmugakonar M, Al-Naemi HA (2019). RAS-mediated oncogenic signaling pathways in human malignancies. Seminars in Cancer Biology.

[6] Hobbs GA, Der CJ (2019). RAS mutations are not created equal. Cancer Discovery.

[7] Simanshu DK, Nissley DV, McCormick F (2017). RAS proteins and their regulators in human disease. Cell.

[8] Resat H, Straatsma TP, Dixon DA, Miller JH (2001). The arginine finger of RasGAP helps Gln-61 align the nucleophilic water in GAP-stimulated hydrolysis of GTP. Proceedings of the National Academy of Sciences of the United States of America.

[9] Scheffzek K, Ahmadian MR, Kabsch W, Wiesmüller L, Lautwein A, Schmitz F, Wittinghofer A (1997). The Ras-RasGAP complex: structural basis for GTPase activation and its loss in oncogenic ras mutants. Science.

[10] Vetter IR, Wittinghofer A (2014). The structure of the G domain of the Ras superfamily. *Ras Superfamily Small G Proteins: Biology and Mechanisms 1: General Features, Signaling* (pp. 25–50).

[11] Kamerlin SCL, Sharma PK, Prasad RB, Warshel A (2013). Why nature really chose phosphate. Quarterly Reviews of Biophysics.

[12] Prasad BR, Plotnikov NV, Lameira J, Warshel A (2013). Quantitative exploration of the molecular origin of the activation of GTPase. Proceedings of the National Academy of Sciences of the United States of America.

[13] Mishra AK, Lambright DG (2016). Invited review: small GTPases and their GAPs. Biopolymers.

[14] Buhrman G, Holzapfel G, Fetics S, Mattos C (2010). Allosteric modulation of Ras positions Q61 for a direct role in catalysis. Proceedings of the National Academy of Sciences of the United States of America.

[15] Xu K, Park D, Magis AT, Zhang J, Zhou W, Sica GL, Ramalingam SS, Curran WJ, Deng X (2019). Small molecule KRAS agonist for mutant KRAS cancer therapy. Molecular Cancer.

[16] Waters AM, Der CJ (2018). KRAS: the critical driver and therapeutic target for pancreatic cancer. Cold Spring Harbor Perspectives in Medicine.

[17] Saliani M, Jalal R, Ahmadian MR (2019). From basic researches to new achievements in therapeutic strategies of KRAS-driven cancers. Cancer Biology and Medicine.

[18] Drosten M, Barbacid M (2020). Targeting the MAPK pathway in KRAS-driven tumors. Cancer Cell.

[19] Ford B, Boykevisch S, Zhao C, Kunzelmann S, Bar-Sagi D, Herrmann C, Nassar N (2009). Characterization of a Ras mutant with identical GDP- and GTP-bound structures. Biochemistry.

[20] Traut TW (1994). Physiological concentrations of purines and pyrimidines. Molecular and Cellular Biochemistry.

[21] Fakih M, O’Neil B, Price TJ, Falchook GS, Desai J, Kuo J (2019). Phase 1 study evaluating the safety, tolerability, pharmacokinetics (PK), and efficacy of AMG 510, a novel small molecule KRAS G12C inhibitor, in advanced solid tumors. Journal of Clinical Oncology.

[22] Papadopoulos KP, Ou S-HI, Johnson ML, Christensen J, Velastegui K, Potvin D (2019). A phase I/II multiple expansion cohort trial of MRTX849 in patients with advanced solid tumors with KRAS G12C mutation. Journal of Clinical Oncology.

[23] First-in-Human Study of JNJ-74699157 in Participants With Tumors Harboring the KRAS G12C Mutation - ClinicalTrials.gov. (n.d.). Retrieved May 4, 2020, from https://clinicaltrials.gov/ct2/show/NCT04006301

[24] A Study of LY3499446 in Participants With Advanced Solid Tumors With KRAS G12C Mutation - ClinicalTrials.gov. (n.d.). Retrieved May 4, 2020, from https://clinicaltrials.gov/ct2/show/NCT04165031

[25] A Study to Test Different Doses of BI 1701963 Alone and Combined With Trametinib in Patients With Different Types of Advanced Cancer (Solid Tumours With KRAS Mutation) - Full Text View - ClinicalTrials.gov. (n.d.). Retrieved May 6, 2020, from https://clinicaltrials.gov/ct2/show/NCT04111458

[26] Hofmann, M. H., Gmachl, M., Ramharter, J., Savarese, F., Gerlach, D., Marszalek, J. R., … Kraut, N. (2019). Abstract PL06-01: discovery of BI-3406: a potent and selective SOS1::KRAS inhibitor opens a new approach for treating KRAS-driven tumors. In *Molecular Cancer Therapeutics* (Vol. 18, pp. PL06-01-PL06-01). American Association for Cancer Research (AACR). 10.1158/1535-7163.targ-19-pl06-01.

[27] Canon J, Rex K, Saiki AY, Mohr C, Cooke K, Bagal D, Gaida K, Holt T, Knutson CG, Koppada N, Lanman BA, Werner J, Rapaport AS, San Miguel T, Ortiz R, Osgood T, Sun JR, Zhu X, McCarter JD, Volak LP, Houk BE, Fakih MG, O’Neil BH, Price TJ, Falchook GS, Desai J, Kuo J, Govindan R, Hong DS, Ouyang W, Henary H, Arvedson T, Cee VJ, Lipford JR (2019). The clinical KRAS(G12C) inhibitor AMG 510 drives anti-tumour immunity. Nature.

[28] Fell, J. B., Fischer, J. P., Baer, B. R., Blake, J. F., Bouhana, K., Briere, D. M., … Marx, M. A. (2020). Identification of the clinical development candidate MRTX849 , a covalent KRAS G12C inhibitor for the treatment of cancer. *Journal of Medicinal Chemistry*, acs.jmedchem.9b02052. 10.1021/acs.jmedchem.9b02052. 10.1021/acs.jmedchem.9b02052 32250617

[29] Prior IA, Lewis PD, Mattos C (2012). A comprehensive survey of Ras mutations in cancer. Cancer Research.

[30] Lohinai Z, Klikovits T, Moldvay J, Ostoros G, Raso E, Timar J, Fabian K, Kovalszky I, Kenessey I, Aigner C, Renyi-Vamos F, Klepetko W, Dome B, Hegedus B (2017). KRAS-mutation incidence and prognostic value are metastatic site-specific in lung adenocarcinoma: poor prognosis in patients with KRAS mutation and bone metastasis. Scientific Reports.

[31] Tímár J (2014). The clinical relevance of KRAS gene mutation in non-small-cell lung cancer. Current Opinion in Oncology.

[32] Ghimessy AK, Gellert A, Schlegl E, Hegedus B, Raso E, Barbai T, Timar J, Ostoros G, Megyesfalvi Z, Gieszer B, Moldvay J, Renyi-Vamos F, Lohinai Z, Hoda MA, Klikovits T, Klepetko W, Laszlo V, Dome B (2019). KRAS mutations predict response and outcome in advanced lung adenocarcinoma patients receiving first-line bevacizumab and platinum-based chemotherapy. Cancers.

[33] Ostrem JM, Peters U, Sos ML, Wells JA, Shokat KM (2013). K-Ras(G12C) inhibitors allosterically control GTP affinity and effector interactions. Nature.

[34] Patricelli MP, Janes MR, Li LS, Hansen R, Peters U, Kessler LV, Chen Y, Kucharski JM, Feng J, Ely T, Chen JH, Firdaus SJ, Babbar A, Ren P, Liu Y (2016). Selective inhibition of oncogenic KRAS output with small molecules targeting the inactive state. Cancer Discovery.

[35] Hunter JC, Manandhar A, Carrasco MA, Gurbani D, Gondi S, Westover KD (2015). Biochemical and structural analysis of common cancer-associated KRAS mutations. Molecular Cancer Research.

[36] Hansen R, Peters U, Babbar A, Chen Y, Feng J, Janes MR, Li LS, Ren P, Liu Y, Zarrinkar PP (2018). The reactivity-driven biochemical mechanism of covalent KRASG12C inhibitors. Nature Structural & Molecular Biology.

[37] Khrenova MG, Kulakova AM, Nemukhin AV (2020). Proof of concept for poor inhibitor binding and efficient formation of covalent adducts of KRAS G12C and ARS compounds. Organic & Biomolecular Chemistry..

[38] Petri, L., Ábrányi-Balogh, É., Imre, T., Pálfy, G., Perczel, A., Knez, D., … Keseru, G. M. (n.d.). Warhead-based cysteine reactivity mapping for optimizing covalent inhibitors. ***Manuscript under review***.

[39] Rachman M, Scarpino A, Bajusz D, Pálfy G, Vida I, Perczel A, Barril X, Keserű GM (2019). DUckCov: a dynamic undocking-based virtual screening protocol for covalent binders. ChemMedChem.

[40] Lito P, Solomon M, Li L-S, Hansen R, Rosen N (2016). Allele-specific inhibitors inactivate mutant KRAS G12C by a trapping mechanism. Science.

[41] Mortier, J., Friberg, A., Badock, V., Moosmayer, D., Schroeder, J., Steigemann, P., et al. (2020). Computationally empowered workflow identifies novel covalent allosteric binders for KRASG12C. *ChemMedChem*, 1–7. 10.1002/cmdc.201900727. 10.1002/cmdc.201900727 PMC7318243 32237114

[42] Janes MR, Zhang J, Li LS, Hansen R, Peters U, Guo X (2018). Targeting KRAS mutant cancers with a covalent G12C-specific inhibitor. Cell.

[43] Shin Y, Jeong JW, Wurz RP, Achanta P, Arvedson T, Bartberger MD, Campuzano IDG, Fucini R, Hansen SK, Ingersoll J, Iwig JS, Lipford JR, Ma V, Kopecky DJ, McCarter J, San Miguel T, Mohr C, Sabet S, Saiki AY, Sawayama A, Sethofer S, Tegley CM, Volak LP, Yang K, Lanman BA, Erlanson DA, Cee VJ (2019). Discovery of N -(1-Acryloylazetidin-3-yl)-2-(1 H -indol-1-yl)acetamides as covalent inhibitors of KRAS G12C. ACS Medicinal Chemistry Letters.

[44] Christensen, J. G., Olson, P., Briere, T., Wiel, C., & Bergo, M. O. (2020). Targeting KRAS G12C -mutant cancer with a mutation-specific inhibitor. Journal of Internal Medicine, (858), 0–2. 10.1111/joim.13057. 10.1111/joim.13057 32176377

[45] Xiong Y, Lu J, Hunter J, Li L, Scott D, Choi HG, Lim SM, Manandhar A, Gondi S, Sim T, Westover KD, Gray NS (2017). Covalent guanosine mimetic inhibitors of G12C KRAS. ACS Medicinal Chemistry Letters.

[46] Lim SM, Westover KD, Ficarro SB, Harrison RA, Choi HG, Pacold ME (2015). Therapeutic targeting of oncogenic K-Ras by a. Angewandte Chemie (International Ed. in English).

[47] Hunter JC, Gurbani D, Ficarro SB, Carrasco MA, Lim SM, Choi HG (2014). In situ selectivity profiling and crystal structure of SML-8-73-1, an active site inhibitor of oncogenic K-Ras G12C. Proceedings of the National Academy of Sciences of the United States of America.

[48] Lim SM, Westover KD, Ficarro SB, Harrison RA, Choi HG, Pacold ME, Carrasco M, Hunter J, Kim ND, Xie T, Sim T, Jänne PA, Meyerson M, Marto JA, Engen JR, Gray NS (2014). Therapeutic targeting of oncogenic K-ras by a covalent catalytic site inhibitor. Angewandte Chemie - International Edition.

[49] Shannon DA, Weerapana E (2015). Covalent protein modification: The current landscape of residue-specific electrophiles. Current Opinion in Chemical Biology.

[50] Weerapana E, Simon GM, Cravatt BF (2008). Disparate proteome reactivity profiles of carbon electrophiles. Nature Chemical Biology.

[51] Los GV, Encell LP, McDougall MG, Hartzell DD, Karassina N, Zimprich C (2008). HaloTag: a novel protein labeling technology for cell imaging and protein analysis. ACS Chemical Biology.

[52] McGregor LM, Jenkins ML, Kerwin C, Burke JE, Shokat KM (2017). Expanding the scope of electrophiles capable of targeting K-Ras oncogenes. Biochemistry.

[53] Niida A, Sasaki S, Yonemori K, Sameshima T, Yaguchi M, Asami T, Sakamoto K, Kamaura M (2017). Bioorganic & medicinal chemistry letters investigation of the structural requirements of K-Ras ( G12D ) selective inhibitory peptide KRpep-2d using alanine scans and cysteine bridging. Bioorganic & Medicinal Chemistry Letters.

[54] Sogabe S, Kamada Y, Miwa M, Niida A, Sameshima T, Kamaura M, Yonemori K, Sasaki S, Sakamoto JI, Sakamoto K (2017). Crystal structure of a human K-Ras G12D mutant in complex with GDP and the cyclic inhibitory peptide KRpep-2d. ACS Medicinal Chemistry Letters.

[55] Maurer T, Garrenton LS, Oh A, Pitts K, Anderson DJ, Skelton NJ, Fauber BP, Pan B, Malek S, Stokoe D, Ludlam MJC, Bowman KK, Wu J, Giannetti AM, Starovasnik MA, Mellman I, Jackson PK, Rudolph J, Wang W, Fang G (2012). Small-molecule ligands bind to a distinct pocket in Ras and inhibit SOS-mediated nucleotide exchange activity. Proceedings of the National Academy of Sciences.

[56] Kessler D, Gmachl M, Mantoulidis A, Martin LJ, Zoephel A, Mayer M, Gollner A, Covini D, Fischer S, Gerstberger T, Gmaschitz T, Goodwin C, Greb P, Häring D, Hela W, Hoffmann J, Karolyi-Oezguer J, Knesl P, Kornigg S, Koegl M, Kousek R, Lamarre L, Moser F, Munico-Martinez S, Peinsipp C, Phan J, Rinnenthal J, Sai J, Salamon C, Scherbantin Y, Schipany K, Schnitzer R, Schrenk A, Sharps B, Siszler G, Sun Q, Waterson A, Wolkerstorfer B, Zeeb M, Pearson M, Fesik SW, McConnell DB (2019). Drugging an undruggable pocket on KRAS. Proceedings of the National Academy of Sciences.

[57] Sun Q, Burke JP, Phan J, Burns MC, Olejniczak ET, Waterson AG, Lee T, Rossanese OW, Fesik SW (2012). Discovery of small molecules that bind to K-Ras and inhibit Sos-mediated activation. Angewandte Chemie - International Edition.

[58] Boriack-Sjodin P a, Margarit SM, Bar-Sagi D, Kuriyan J (1998). The structural basis of the activation of Ras by Sos. Nature.

[59] Cruz-Migoni A, Canning P, Quevedo CE, Bataille CJR, Bery N, Miller A, Russell AJ, Phillips SEV, Carr SB, Rabbitts TH (2019). Structure-based development of new RAS-effector inhibitors from a combination of active and inactive RAS-binding compounds. Proceedings of the National Academy of Sciences of the United States of America.

[60] Quevedo CE, Cruz-Migoni A, Bery N, Miller A, Tanaka T, Petch D, Bataille CJR, Lee LYW, Fallon PS, Tulmin H, Ehebauer MT, Fernandez-Fuentes N, Russell AJ, Carr SB, Phillips SEV, Rabbitts TH (2018). Small molecule inhibitors of RAS-effector protein interactions derived using an intracellular antibody fragment. Nature Communications.

[61] Tran, T. H., Alexander, P., Dharmaiah, S., & Agamasu, C. (2020). The small molecule BI-2852 induces a nonfunctional dimer of KRAS.*, 117*(7), 3363–3364. 10.1073/pnas.1918164117. 10.1073/pnas.1918164117 PMC7035607 32047043

[62] Guillard S, Kolasinska-Zwierz P, Debreczeni J, Breed J, Zhang J, Bery N, Marwood R, Tart J, Overman R, Stocki P, Mistry B, Phillips C, Rabbitts T, Jackson R, Minter R (2017). Structural and functional characterization of a DARPin which inhibits Ras nucleotide exchange. Nature Communications.

[63] Shin S, Choi D, Jung K, Bae J, Kim J, Park S (2017). Antibody targeting intracellular oncogenic Ras. Nature Communications.

[64] Shin, S. M., Choi, D. K., Jung, K., Bae, J., Kim, J. S., Park, S. W., Song, K. H., & Kim, Y. S. (2017). Antibody targeting intracellular oncogenic Ras mutants exerts anti-tumour effects after systemic administration. *Nature Communications, 8*. 10.1038/ncomms15090. 10.1038/ncomms15090 PMC5436137 28489072

[65] Bermingham A, Choy TJ, Cregg JJ, Gill AL, Goldsmith MA, Hansen RL (2019). Inhibition of the oncogenic, GTP-bound form of KRASG12C by second generation.

[66] Shima F, Yoshikawa Y, Ye M, Araki M, Matsumoto S, Liao J, Hu L, Sugimoto T, Ijiri Y, Takeda A, Nishiyama Y, Sato C, Muraoka S, Tamura A, Osoda T, Tsuda KI, Miyakawa T, Fukunishi H, Shimada J, Kumasaka T, Yamamoto M, Kataoka T (2013). In silico discovery of small-molecule Ras inhibitors that display antitumor activity by blocking the Ras-effector interaction. Proceedings of the National Academy of Sciences of the United States of America.

[67] Feng H, Zhang Y, Bos PH, Chambers JM, Dupont MM, Stockwell BR (2019). K-RasG12D has a potential allosteric small molecule binding site. Biochemistry.

[68] Bery, N., Legg, S., Debreczeni, J., Breed, J., Embrey, K., Stubbs, C., Kolasinska-Zwierz P., Barrett N., Marwood R., Watson J., Tart J., Overman R., Miller A., Phillips C., Minter R. Rabbitts, T. H. (2019). KRAS-specific inhibition using a DARPin binding to a site in the allosteric lobe. Nature Communications, 10(1), 0–10. 10.1038/s41467-019-10419-2, 2607. 10.1038/s41467-019-10419-2 PMC6565726 31197133

[69] Margarit SM, Sondermann H, Hall BE, Nagar B, Hoelz A, Pirruccello M, Bar-Sagi D, Kuriyan J (2003). Structural evidence for feedback activation by Ras·GTP of the Ras-specific nucleotide exchange factor SOS. Cell.

[70] Jeng H-H, Taylor LJ, Bar-Sagi D (2012). Sos-mediated cross-activation of wild-type Ras by oncogenic Ras is essential for tumorigenesis. Nature Communications.

[71] Burns MC, Sun Q, Daniels RN, Camper D, Kennedy JP, Phan J, Olejniczak ET, Lee T, Waterson AG, Rossanese OW, Fesik SW (2014). Approach for targeting Ras with small molecules that activate SOS-mediated nucleotide exchange. Proceedings of the National Academy of Sciences.

[72] Hodges TR, Abbott JR, Little AJ, Sarkar D, Salovich JM, Howes JE, Akan DT, Sai J, Arnold AL, Browning C, Burns MC, Sobolik T, Sun Q, Beesetty Y, Coker JA, Scharn D, Stadtmueller H, Rossanese OW, Phan J, Waterson AG, McConnell DB, Fesik SW (2018). Discovery and structure-based optimization of benzimidazole-derived activators of SOS1-mediated nucleotide exchange on RAS. Journal of Medicinal Chemistry.

[73] Hillig RC, Sautier B, Schroeder J, Moosmayer D, Hilpmann A, Stegmann CM, Werbeck ND, Briem H, Boemer U, Weiske J, Badock V, Mastouri J, Petersen K, Siemeister G, Kahmann JD, Wegener D, Böhnke N, Eis K, Graham K, Wortmann L, von Nussbaum F, Bader B (2019). Discovery of potent SOS1 inhibitors that block RAS activation via disruption of the RAS–SOS1 interaction. Proceedings of the National Academy of Sciences of the United States of America.

[74] Evelyn CR, Duan X, Biesiada J, Seibel WL, Meller J, Zheng Y (2014). Rational design of small molecule inhibitors targeting the Ras GEF, SOS1. Chemistry and Biology.

[75] Winter JJG, Anderson M, Blades K, Brassington C, Breeze AL, Chresta C, Embrey K, Fairley G, Faulder P, Finlay MRV, Kettle JG, Nowak T, Overman R, Patel SJ, Perkins P, Spadola L, Tart J, Tucker JA, Wrigley G (2015). Small molecule binding sites on the Ras:SOS complex can be exploited for inhibition of Ras activation. Journal of Medicinal Chemistry.

[76] Manuscript, A. (2011). NIH public access, (12), 1693–1713.

[77] Lampson BL, Pershing NLK, Prinz JA, Lacsina JR, Marzluff WF, Nicchitta CV, MacAlpine DM, Counter CM (2013). Rare codons regulate KRas oncogenesis. Current Biology.

[78] Pershing NLK, Lampson BL, Belsky JA, Kaltenbrun E, MacAlpine DM, Counter CM (2015). Rare codons capacitate Kras-driven de novo tumorigenesis. Journal of Clinical Investigation.

[79] Chi S, Kitanaka C, Noguchi K, Mochizuki T, Nagashima Y, Shirouzu M, Fujita H, Yoshida M, Chen W, Asai A, Himeno M, Yokoyama S, Kuchino Y (1999). Oncogenic Ras triggers cell suicide through the activation of a caspase-independent cell death program in human cancer cells. Oncogene.

[80] Lv C, Hong Y, Miao L, Li C, Xu G, Wei S, Wang B, Huang C, Jiao B (2013). Wentilactone A as a novel potential antitumor agent induces apoptosis and G2/M arrest of human lung carcinoma cells, and is mediated by HRas-GTP accumulation to excessively activate the Ras/Raf/ERK/p53-p21 pathway. Cell Death and Disease.

[81] Burns MC, Sun Q, Daniels RN, Camper D, Kennedy JP, Phan J, Olejniczak ET, Lee T, Waterson AG, Rossanese OW, Fesik SW (2014). Approach for targeting Ras with small molecules that activate SOS-mediated nucleotide exchange. Proceedings of the National Academy of Sciences of the United States of America.

[82] Burns MC, Howes JE, Sun Q, Little AJ, Camper DMV, Abbott JR, Phan J, Lee T, Waterson AG, Rossanese OW, Fesik SW (2018). High-throughput screening identifies small molecules that bind to the RAS:SOS:RAS complex and perturb RAS signaling. Analytical Biochemistry.

[83] Nyíri, K., Koppány, G., Tímár, J., Tóvári, J., Kígyós, A., Ranđelović, I., … Vértessy G., B. (2020). Method and apparatus to facilitate the binding of the GAP protein to the mutant RAS protein by molecular agents to cure RAS-mutation related cancers. EP20020099.

[84] Nagasaka M, Li Y, Sukari A, Ou SHI, Al-Hallak MN, Azmi AS (2020). KRAS G12C game of thrones, which direct KRAS inhibitor will claim the iron throne?. Cancer Treatment Reviews.

[85] Caruso, C., & Rose, S. (2020). Dueling KRASG12C inhibitors achieve responses. *Cancer discovery, 10*(1). 10.1158/2159-8290.CD-ND2019-012. 10.1158/2159-8290.CD-ND2019-012 31822538

[86] Xue JY, Zhao Y, Aronowitz J, Mai TT, Vides A, Qeriqi B, Kim D, Li C, de Stanchina E, Mazutis L, Risso D, Lito P (2020). Rapid non-uniform adaptation to conformation-specific KRAS(G12C) inhibition. Nature.

[87] Hata AN, Shaw AT (2020). Resistance looms for KRASG12C inhibitors. Nature Medicine.

[88] Lou K, Steri V, Ge AY, Hwang YC, Yogodzinski CH, Shkedi AR, Choi ALM, Mitchell DC, Swaney DL, Hann B, Gordan JD, Shokat KM, Gilbert LA (2019). KRASG12C inhibition produces a driver-limited state revealing collateral dependencies. Science Signaling.

[89] Yaeger R, Solit DB (2020). Overcoming adaptive resistance to KRAS inhibitors through vertical pathway targeting. Clinical Cancer Research.

[90] Hallin J, Engstrom LD, Hargi L, Calinisan A, Aranda R, Briere DM (2020). The KRASG12C inhibitor MRTX849 provides insight toward therapeutic susceptibility of KRAS-mutant cancers in mouse models and patients. Cancer Discovery.

[91] Nichols R, Schulze C, Bermingham A, Choy T, Cregg J, Kiss G, Marquez A, Reyes D, Saldajeno-Concar M, Weller C, Whalen D, Yang Y, Wang Z, Koltun ES, Singh M, Wildes D, Gill AL, Hansen R, Kelsey S, Goldsmith M, Smith J (2020). A06 tri-complex inhibitors of the oncogenic, GTP-bound form of KRASG12C overcome RTK-mediated escape mechanisms and drive tumor regressions in preclinical models of NSCLC. Journal of Thoracic Oncology.

