# Supplementary material for: Structure-based inhibitor design of mutant RAS proteins—a paradigm shift
Source: Cancer Metastasis Rev. 2020 Jul 26;39(4):1091–105. doi: 10.1007/s10555-020-09914-6 (PMC7680331; doi:10.1007/s10555-020-09914-6)
Supplement: Supplementary file 1 — (PDF 518 kb) [file 10555_2020_9914_MOESM1_ESM.pdf]

Supplementary Material to the article:

Structure based inhibitor design of mutant RAS proteins – a paradigm shift

Kinga Nyíri [1,2,*,†], Gergely Koppány [1,2,†] and Beáta G. Vértessy [1,2,*]

[1] Department of Applied Biotechnology and Food Sciences, Budapest University of Technology and Economics, Budapest 1111, Hungary

[2] Institute of Enzymology, Research Centre for Natural Sciences, Hungarian Academy of Sciences, Budapest 1117, Hungary

* Correspondence: knyiri@mail.bme.hu (K.N.); vertessy@mail.bme.hu (B.G.V.)

† These authors contributed equally to this paper.

Supplementary Figure S1

Supplementary Figure S2

Supplementary Figure S3

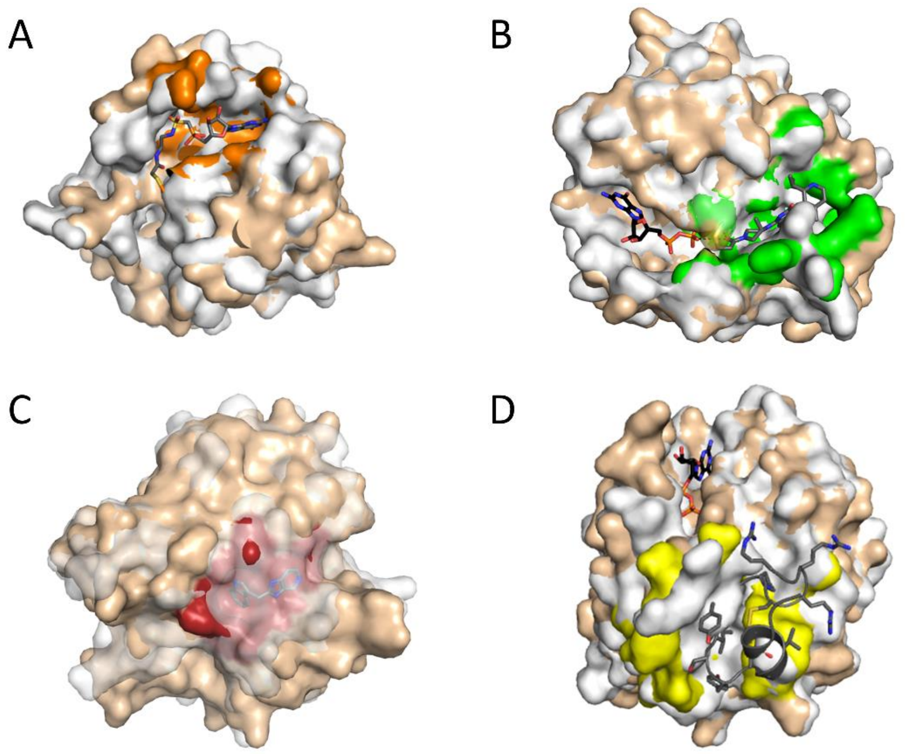

Supplementary Figure S1. Formation of binding pockets on KRAS.
Superimposition GDP-bound KRAS (white, PDB ID: 5W22) with **A**) S7-bound KRAS (wheat and green, PDB ID: 5F2E), **B**) S12-bound KRAS (wheat and orange, PDB ID: 5KYK), **C**) peptide-bound KRAS (wheat and yellow, PDB ID: 5XCO), **D**) S3-bound KRAS (wheat and ruby, PDB ID: 4EPV) both in surface representation. Figure was made by using PyMOL Molecular Graphics System.

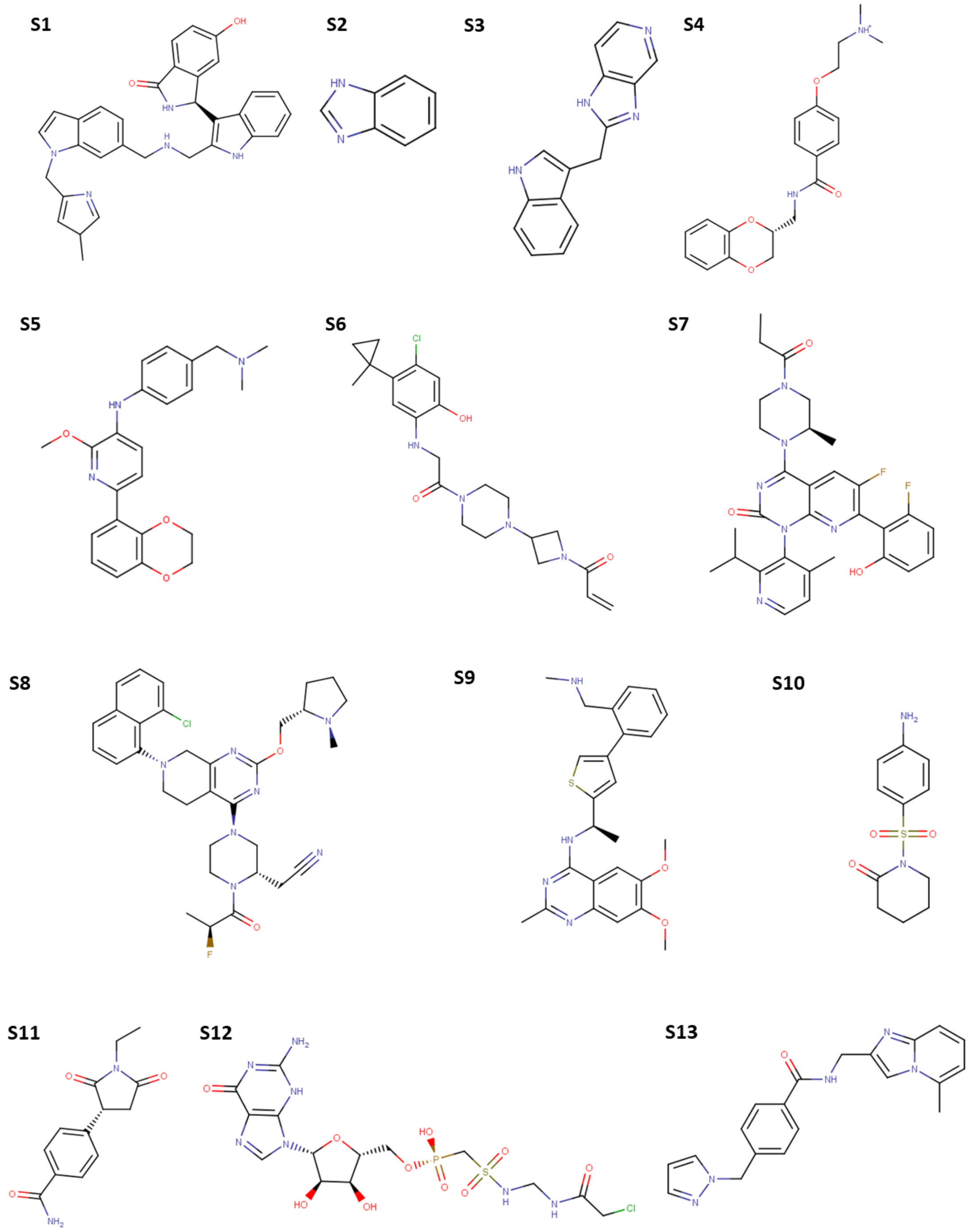

Supplementary Figure S2. Chemical formulae of compounds S1-S13, listed in Table 1

S1) BI-2852 [56], S2) Benzimidazole [55], S3) 'Compound -4' [57], S4) ABD-4, S5) ABD-7 [60], S6) ARS-853 [34], S7) AMG-510 [27], S8) MRTX849 [28], S9) BAY-293[73], S10) Small molecule compound that binds to the HRAS-SOS interface, S11) Covalent inhibitor targeting Cys-118 of HRAS [75], S12) XY-02-075 a covalent GTP analogue, targeting Cys-12 [45], S13) 'Compound-5', stabilizing molecule of the KRAS-GAP complex.

A

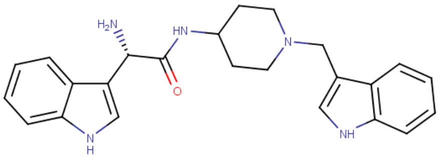

B

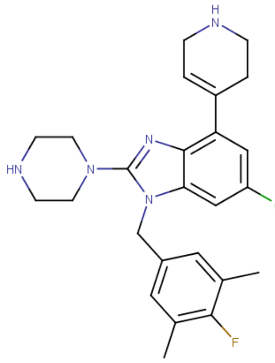

Supplementary Figure S3. –Representative compounds enhancing RAS-SOS interaction
A) Compound from the structure PDB ID: 4NYM [56] **B**) Compound from the structure PDB ID: 6D5G [57]
